# Manikins versus simulated patients in emergency medicine training: a comparative analysis

**DOI:** 10.1007/s00068-021-01695-z

**Published:** 2021-07-30

**Authors:** Jasmina Sterz, Niklas Gutenberger, Maria-Christina Stefanescu, Uwe Zinßer, Lena Bepler, Svea Linßen, Verena Schäfer, Patrick Carstensen, René Danilo Verboket, Farzin Adili, Miriam Ruesseler

**Affiliations:** 1 Department for Trauma-, Hand- and Reconstructive Surgery, University Hospital Frankfurt, Goethe University, Theodor-Stern-Kai 7, Frankfurt, Germany; 2 Medical Faculty, Frankfurt Interdisciplinary Simulation Center FIneST, Goethe University, Frankfurt, Germany; 3 Medical Faculty, SP Training Center, Goethe University, Frankfurt, Germany; 4 Division of Vascular and Endovascular Surgery, Department of Vascular Medicine, Klinikum Darmstadt, Darmstadt, Germany

**Keywords:** Simulated patients, Patient manikins, Simulation training, Undergraduate medical education

## Abstract

**Purpose:**

Every physician must be able to sufficiently master medical emergencies, especially in medical areas where emergencies occur frequently such as in the emergency room or emergency surgery. This contrasts with the observation that medical students and young residents often feel insufficiently prepared to handle medical emergencies. It is therefore necessary to train them in the treatment of emergency patients. The aim of this study is to analyze the influence of the assignment of manikin versus simulated patients during a training for undergraduate medical students on learning outcomes and the perceived realism.

**Methods:**

The study had a prospective cross-over design and took place in a 3-day emergency medicine training for undergraduate medical students. Students completed three teaching units (‘chest pain’, ‘impaired consciousness’, ‘dyspnea’), either with manikin or simulated patient. Using a questionnaire after each unit, overall impression, didactics, content, the quality of practical exercises, and the learning success were evaluated. The gained competences were measured in a 6-station objective structured clinical examination (OSCE) at the end of training.

**Results:**

126 students participated. Students rated simulated patients as significantly more realistic than manikins regarding the possibility to carry out examination techniques and taking medical history. 54.92% of the students would prefer to train with simulated patients in the future. Regarding the gained competences for ‘chest pain’ and ‘impaired consciousness’, students who trained with a manikin scored less in the OSCE station than the simulated patients-group.

**Conclusion:**

Simulated patients are rated more realistic than manikins and seem to be superior to manikins regarding gained competence.

## Introduction

Every physician must be able to sufficiently master medical emergencies. Appropriate implementation of the recommended Algorithms e.g. such as ATLS (Advanced Trauma Life Support) or Prehospital trauma life support (PHTLS) for traumatized patients or BLS/ALS (Basic/Advanced Life Support) during resuscitation significantly improves the survival rate of patients with trauma or with cardiac arrest, respectively [1–5]. This contrasts to the oberservation that young residents often do not feel able capable to cope with the emergency care of patients in life-threatening situations [6, 7].

In traditional teaching formats such as traditional bedside teaching, it is hardly possible to teach and train emergency medical and surgical skills in a structured and planned manner. Supported by strong scientific evidence, simulation-based medical education (SBME) offers a valuable approach to acquire practical management skills for the sake of effective learning [8]. These benefits become evident in teaching emergency medical skills. Ruesseler et al. were able to demonstrate that SBME improves student performance in identifying and managing medical emergencies [9]. Furthermore, simulated emergency scenarios may induce stress levels in students that are comparable to the stress levels which medical doctors display in real-life situations [10]. Moreover, the incorporation of in situ simulation trainings correlates with an improved patient morbidity and mortality [11, 12]. The assessed performance in simulated resuscitations remarkably approximates the performance of real resuscitation scenarios at clinical workplaces [13].

The benefit of using manikins in medical education has been proven in many studies, both with regard to the learning effect and the acceptance among the trainees [14–16]*.* However, the degree of realism which is required from the manikins to facilitate the learning process has not yet been fully established. In a study by Berkenstadt et al., experts evaluated the use of a trauma simulator as a tool for chest drain insertion during an ATLS course. These experts found the various steps required for chest drain insertion to be similar to the human’s equivalent and recommended the use of this simulator for training novice doctors in chest drain insertion [17]. Massoth et al. demonstrated that the use of high-fidelity manikins in an advanced life support training for undergraduate medical students as compared to low-fidelity manikins, did not result in an improved performance, but induced undesirable side effects such as overconfidence [18].

The utilization of simulated patients (SPs) represents an alternate approach to SBME. As defined by Cleland et al. SPs ‘play roles’, which means they simulate 'real' patients. For doing this, these specially trained actresses take on the role of patients and other actors in the healthcare system to support exercises and examination scenarios in medical teaching [19]. SP-programs are implemented at every medical school in German-speaking countries [20]. A review published in 2017 ‘found evidence in support of positive knowledge and behavior change in learners’ [21].

However, invasive procedures such as endotrachael intubation or insertion of central venous lines cannot be practiced on SPs, not to speak of pathological vital signs and symptoms which can only be simulated to a limited extent. Novel simulation monitor technologies such as the ALSi^®^ (American 3B Scientific, Tucker, GA, USA) allow for the display of pathological vital signs on a monitor which is directly connected to the SP.

Although manikins and SPs alike present well-established methods of medical education, there is still a lack of scientific evidence when to utilize one or the other and which approach proves ultimately to be superior. Wisborg et al. analyzed the use of patient manikins and SPs in simulations for trauma teams and found no differences with regard to the perception of learning success, realism and the feeling of embarrassment [22]. To our knowledge, no robust comparative data exist with regard to the teaching of emergency skills and procedures in undergraduate medical education.

Therefore, the present study was devised to investigate learning outcomes and perceived realism of undergraduate emergency training using manikins versus SP.

## Methods

### Trial design

A prospective cross-over study was designed according to the rules of the Ethics Board at Goethe University Medical School Frankfurt, Germany, no Ethics Approval was required for conducting this study. Still, the study was conducted according to ethical principles of the Helsinki Declaration (Ethical Principles for Medical Research Involving Human Subjects).

### Participants

Study participants were fourth or fifth year undergraduate medical students. Participation in the study was voluntary and took place after written informed consent. Basic data regarding student age, sex, previous experience in emergency medicine and duration of previous study were collected using a questionnaire.

### Study protocol

The students participated in a three-day mandatory practical skills course as part of their emergency medicine curriculum. In this setup, students first completed four modules in small groups of 6 students, in which they acquired basic emergency medicine skills, e.g., airway management, performing the steps of Basic Life Support, performing the ABCDE to initially assess critically ill or traumatized patients. During the following two days, the students completed four teaching units leading symptoms that are common in emergency medicine and trauma/emergency surgery: chest pain, dyspnea, impaired consciousness, cardiac arrest.

In each of these units, the students first devised a diagnosis and treatment algorithm for the most common differential diagnoses before undergoing two complete practical scenarios as a medical team. At the conclusion of each unit, a structured debriefing took place. The training sessions were led by two instructors: an emergency medicine physician and a peer tutor.

**Fig. 1:**
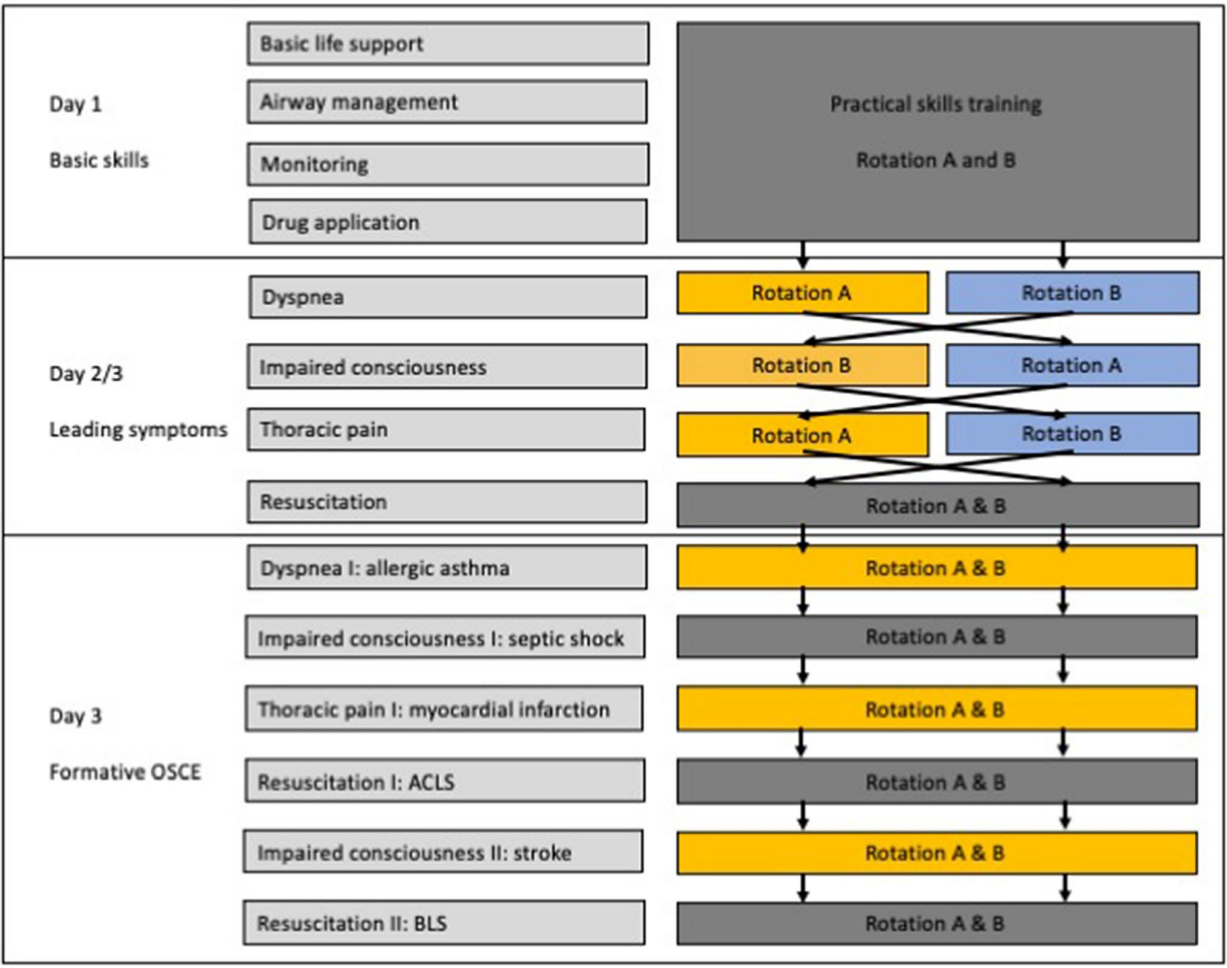
Teaching units of the course and students’ rotation. Teaching units that are not part of the study are colored in dark gray, teaching units with manikins are colored in blue, teaching units with SPs are colored in orange

The entire training ended with a formative OSCE (Objective structured clinical examination), in which a team of two completed a total of six scenarios and got a final feedback (Fig. 1).

All students who completed the training, were randomly allocated to groups of six by the Department of Students’ Administration of Goethe-University. Neither the authors nor participation in the study had any influence on this allocation.

As part of the study, the students were randomized to either rotation A or rotation B and completed the leading symptom modules 'Dyspnea’, 'Chest pain' and ‘Impaired consciousness' in a cross-over design either with a manikin or an SP (Fig. 1), thus every group of students trained with both SPs and manikin. Since it was not possible to perform a cardio-pulmonary resuscitation on a living person (SP), this module was excluded from the present study.

For the manikin training, Resusci–Anne manikins from Laerdal (Laerdal Medical, Stavanger, Norway) were used. Patient communication, as the patient answers questions regarding medical history, comments procedures, etc. was performed by the trainer and his tutor based on a detailed role script. For this, both received a detailed training as described for the SP.

In the SP-cohort, amateur actors had to follow a detailed role script, which was identic to the manikin role script and received a personal acting training. As part of the training, the trainer queried the previously learned role script and ambiguous content and emerging affects as well as upcoming emotions were made subject to discussion. Rules of conduct such as dealing with open questions or insecure students were explained and practiced. Depending on the level of previous experience of the SP, the basic training lasted 1–2 h. Whenever needed, the SPs had professional make-up for their role (e.g., blue lips for cyanosis, petechiae for sepsis, etc.).

### Evaluation

Every teaching unit was evaluated using an anonymized online questionnaire (SurveyMonkey Europe UC, Dublin, Ireland) which consisted of 15 items. Students were asked to rate the overall impression, didactics, content, the quality of practical exercises and the learning success on a 6-point Likert scale (1 = very good to 6 = insufficient). Eight items asked for the perception of reality and were rated on a 6-point Likert scale (from 1 = total disagree to 6 = total agree). The perceived stress level was rated on a visual analog scale ranging from 0 = no stress to 10 = greatest imaginable stress, which is an useful instrument for measuring differences in stress perception between two groups [23].

After completion of all modules and prior to the formative OSCE, the participants underwent a final evaluation, which was devised to compare different levels of perceived reality during physical examination, medical history survey and performing procedural skills. For this purpose, the students were asked to evaluate three items regarding the realism of examination, anamnesis and practical skills on a 6-point Likert scale (from 1 = total disagree to 6 = total agree) twice: first, when they trained with manikins and secondly during the interaction with SPs. Finally, the students were asked which of the teaching media they would prefer for further trainings.

### Formative OSCE assessment

The formative OSCE assessment consisted of six stations, from which three were part of the present study: one dealing with a patient suffering an ongoing asthmatic attack, one patient suffering a myocardial infarction and the third one with a patient suffering a stroke. The given time frame for each station was 5 min followed by 2 min structured feedback. Students completed the stations in a team of two.

The students were rated on a well-defined checklist assessing their competences in the diagnostic and therapy algorithm as well as in communication with their team member and the patient. Afterward, the students received a short feedback session regarding their performance, including suggestions for improvement.

### Statistical analysis

The data were entered in Excel (Microsoft Inc., Redmond, WA, USA), the further statistical analysis was carried out with SPSS (SPSS Inc., Chicago, IL, USA).

A two-sided *t*-test was used to compare the learning success of the students based on a normal distribution. The results of the evaluation of the individual teaching units, the questionnaire on the stress level and the final evaluation were analyzed using the Mann–Whitney *U* test for non-parametric data. The influence of gender on learning success was investigated using linear regression analyzes.

The free comments were analyzed according to the principles of qualitative content analysis [24].

## Results

126 students participated in the study. On average, the participants were 25.3 years (min = 22 years; max = 45 years) old, 77 participants were female (61.1%).

### Results of the formative OSCE assessment

**Fig. 2:**
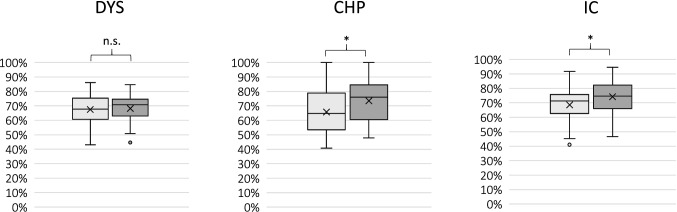
Results in the formative OSCE in the three modules DYS (dyspnea), CHP (chest pain) and IC (impaired consciousness). *p ≤ 0.05; **p ≤ 0.01; n.s. = *p* ≥ 0.05

In the teaching unit ‚chest pain’ and ‘impaired consciousness’, students who trained with a manikin scored significantly less than the group who trained with a SP. In the teaching unit ‘dyspnea’, no significant differences regarding the results between the two groups were found (see also Fig. 2).

### Evaluation of the teaching units

Table 1a-c shows the results of the evaluation of the teaching units. The unit ‘chest pain’ was rated significantly more positive by students who trained with SPs as compared to manikins (*p* ≤ 0.01). Likewise, in this module content (*p* ≤ 0.01), didactics (*p* ≤ 0.01) and the quality of the practical exercises (*p* ≤ 0.01) received higher ratings in the SP group. No statistically different results between SP and manikins were obtained with regard to the learning success *(p* = 0.05); there were also no differences in the teaching units ‘dyspnea’ and ‚impaired consciousness'.

**Table 1:** Evaluation of the unit (a) ‘dyspnea’, (b) ‘chest pain’, (c) ‘Impaired consciousness’

	Sim	SP	*p*
(a)			
Overall	1.4 (± 0.75)	1.6 (± 0.73)	0.26
Content	1.3 (± 0.53)	1.5 (± 0.53)	0.1
Didactics	1.3 (± 0.64)	1.5 (± 0.56)	0.08
Practical exercises	1.3 (± 0.63)	1.3 (± 0.5)	0.98
Learning success	1.4 (± 0.61)	1.5 (± 0.62)	0.28
(b)			
Overall	1.6 (± 0.59)	1.3 (± 0.53)	0.002
Content	1.4 (± 0.56)	1.1 (± 0.35)	0.02
Didactics	1.4 (± 0.56)	1.1 (± 0.35)	0.002
Practical exercises	1.4 (± 0.69)	1.1 (± 0.37)	0.02
Learning success	1.4 (± 0.62)	1.2 (± 0.39)	0.05
(c)			
Overall	1.5 (± 0.59)	1.3 (± 0.52)	0.12
Content	1.3 (± 0.48)	1.3 (± 0.51)	0.58
Didactics	1.3 (± 0.44)	1.4 (± 0.6)	0.53
Practical exercises	1.4 (± 0.57)	1.2 (± 0.42)	0.11
Learning success	1.4 (± 0.55)	1.4 (± 0.55)	0.78

Ratings are shown as Mean ± SD

### Perception of reality

**Fig. 3:**
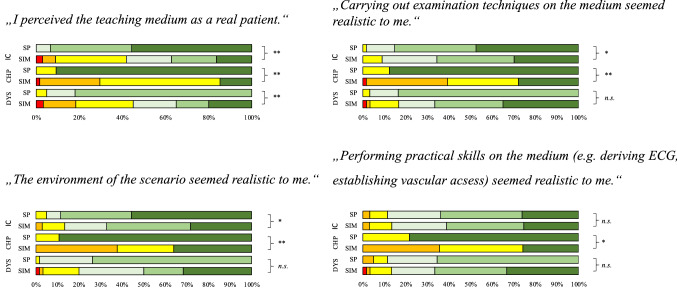
Perception of reality of SP versus manikins in the three modules DYS (dyspnea), CHP (chest pain) and IC (impaired consciousness) for 4 evaluation statements. Rating took place on a 6-point Likert scale with red square– strongly disagree, orange square– disagree, yellow square– slightly disagree, white square– slightly agree, light green square– agree, dark green square strongly agree. *p ≤ 0.05; **p ≤ 0.01; n.s. = *p* ≥ 0.05

In the teaching unit ‚chest pain’ the consent to all statements regarding the perceived reality was significantly higher in the SP group, whereas in the teaching unit, ‘Dyspnea’ only the consent regarding the statement ‘I perceived the teaching medium as a real patient’ was higher when students trained with a SP. In the teaching unit, ‘impaired consciousness’ results were equivocal: half of the items were rated higher when students trained with SPs, whereas no differences were found in the other items. Figure 3 depicts the perception of reality for each teaching unit.

### Perceived stress level

The self-assessment of the stress level showed no significant differences between the groups. The mean stress level was 5.5 ± 1.97. Table 2 shows the perceived stress level of the teaching units.

**Table 2:** Perceived stress level

	Manikins	SP	*p*
Dyspnea	5.53 ± 1.81	5.98 ± 1.79	0.19
Chest pain	5.57 ± 2.12	6.01 ± 1.97	0.19
Impaired consciousness	4.94 ± 1.96	5.0 ± 1.90	0.86

Ratings are shown as Mean ± SD

### Final evaluation

**Fig. 4:**
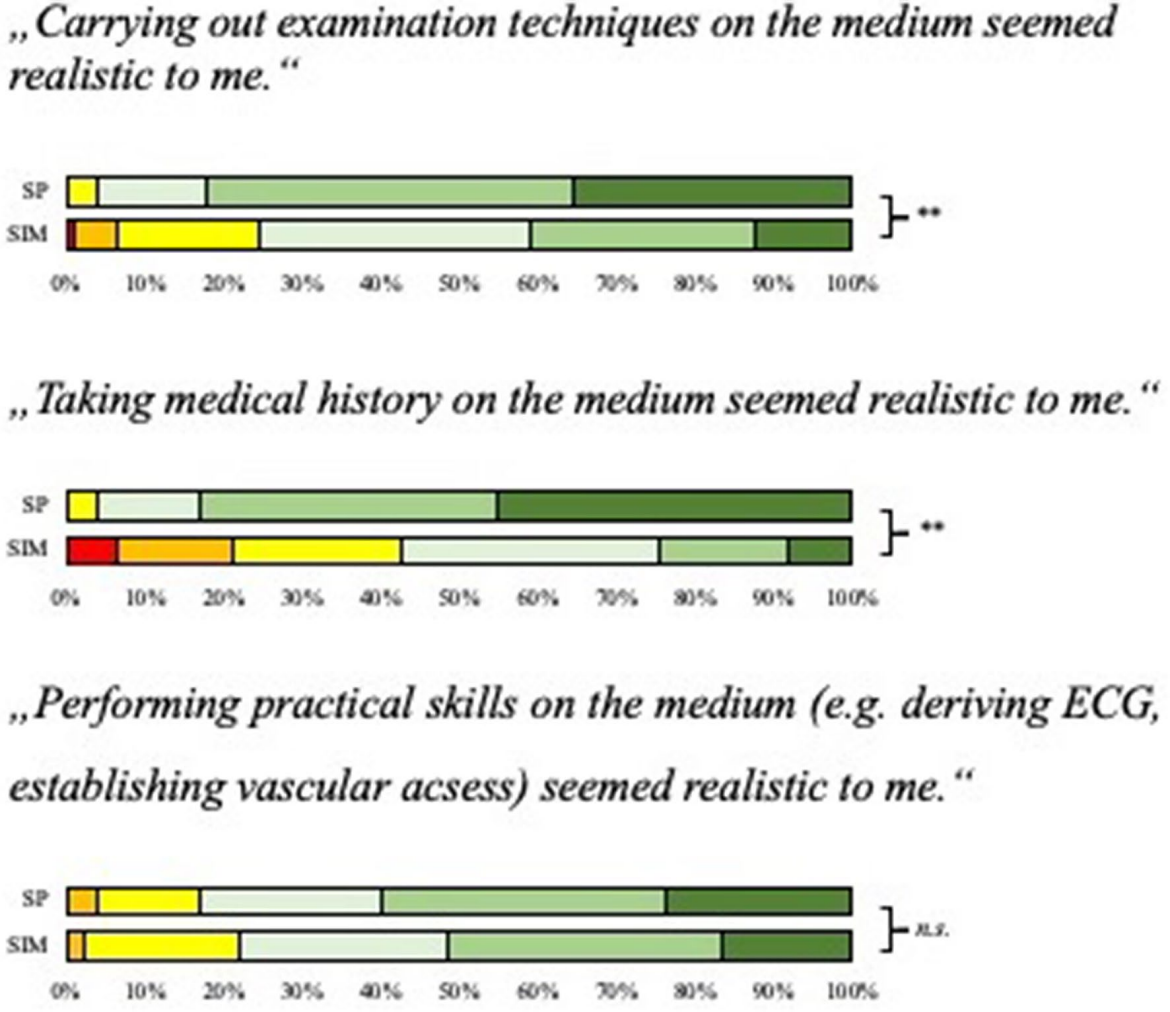
Results of the final evaluation after the course red square–strongly disagree, orange square–disagree, yellow square–slightly disagree, white square–slightly agree, light green square–agree, dark green square–strongly agree. *p ≤ 0.05; **p ≤ 0.01; n.s. = *p* ≥ 0.05

In the final evaluation, students rated SPs as significantly more realistic than manikins regarding the possibility to carry out examination techniques and taking medical history (see also Fig. 4). Regarding the possibility to perform practical skills like establishing a vascular access, there were no differences between the evaluation of SPs and manikins. Overall, 54.9% of the students would prefer to train with SPs in following trainings and 12.3% would prefer to train with manikins. 32.8% of the students stated, that they do not prefer one of the teaching media.

### Cost analysis

The costs of using a SP amounted € 15 per hour. For the present study, SP’s were deployed in the three teaching units ‘dyspnea’, ‘chest pain’, ‘impaired consciousness’: each requiring an SP for 3 h. During each course, each unit had to be repeated 6 times as 6 groups of students were trained, resulting in a total of 54 h of SP per course with 36 students (costs per course € 810; costs per student € 22,50). With 5 courses per semester, this sums to a total cost of € 4050 per semester when using simulated patients.

In contrast, a manikin like the one used in the present study costs approximately € 10.500 (Resusci Anne QCPR AW Torso plus one iv-arm, one measurement arm, two legs and one SimPad PLUS SkillReporter, Retrieved on April 2nd, 2020 from laerdal.com/de). Based on our experience, carrying out 5 courses per semester, there are costs for disposals and maintenance of approx. € 1300 per manikin (e.g., new veins, new skin) and per semester. Because several modules have to take place in parallel, a total of four manikins would be required. The costs for disposables e.g. ECG-electrodes or peripheral venous catheter do not differ between the teaching media.

## Discussion

In the present study, perceived realism, teaching efficacy and inherent costs of SP’s and manikins as part of an undergraduate emergency medicine curriculum were analyzed. Most students rated SPs as more realistic than manikins and in all teaching units, significant more students agreed to the statement “I perceived the SP as a real patient”. These data corroborate the results of other international studies which demonstrated that interactions with SPs come close to real doctor-patient encounters [25–27]. In addition, the scenario and circumstances in which the teaching units took place‚ was perceived as remarkably realistic. This may be indicative of a strong immersion of the trainees in the simulation while interacting with simulated patients.

Regardless of the teaching medium, the subjective stress levels of the participants were moderate in both groups. However, while the present study only determined subjective stress perception for this study, others also gaged cortisol and alpha-amylase levels during emergency simulations with high-fidelity manikins or simulated patients [28]. Yet, both parameters were not indicative for differences in the stress response of the trainees. This corresponds to the results of the present study. A moderate level of stress appears to be important for learning: under a certain level of stress, student performance can improve [29], but excessive stress can also have a negative impact on performance [30]. Therefore, manikins and simulated patients alike seem to be equivalent based on the results of the present study.

In the present study, students who trained with simulated patients outperformed those students who trained with manikins in two of three teaching units. So far, there is little data to compare the influence of SPs and manikins on the teaching success. For example, Ignatico et al. found no significant differences in the performance between nursing students that trained with a SP or with a manikin in deteriorating patient simulations [31]. In contrast to this, Tuzer et al. compared the effects of using manikins and SPs on the thorax-, lung-, and cardiac examination skills of undergraduate nursing students. In this study, students who trained with SPs achieved higher knowledge scores than those students who trained with manikins. Nonetheless, the technical performance of the skill was not different between the groups [32]. We hypothesize, that a possible increase of theoretical knowledge may have also played a role in our 'chest pain' group which favored SPs. For mastering the respective OSCE station, a great deal of theoretical knowledge was necessary, in particular choosing the correct drug medication and dosages.

The results of the present study show that only 12.3% of the students preferred to train with manikins in the future. This was in contrast to others, who compared the use of SPs and manikins during a trauma simulation and found no general preference for either. Nevertheless, they observed a tendency toward SP’s if the patient is supposed to talk and interact with the trauma team [22]. One possible reason for this is always an indirect, non-verbal communication between humans that cannot be simulated using a manikin. Then again, Gilett et al. report that the trainees participating in a mass casualty drill preferred training with manikins [33]. The ‘patients’ in this training were severely wounded and suffered from severe injuries such as amputations. Those injuries cannot be simulated with SP’s. The scenario required invasive measures e.g., the placement of a thoracic drainage. Therefore, the choice of the respective teaching medium needs to suite the simulation objective and it is not possible to make an absolute statement about the general superiority of one of these teaching medium regarding all teaching environments and for all learning objectives.

There are some limitations in the present study. During the formative OSCE assessment, only simulated patients were used. Because of this, one factor that could have influenced the results in the formative OSCE in favor of the students who trained with SPs could be, that they already were used to this medium. Nevertheless, the authors deliberately decided to use only SPs in the OSCE for two reasons: First, due to the cross-over design of the study, every student trained with SPs and manikins during the training. Because of this, it can be assumed that all students were used to both teaching media. Second, to make the results in the formative OSCE comparable between the groups, it was necessary to compare the groups in exactly the same setting.

In this study, it was not examined, how far the training with SP’s and manikins affected real student-patient interactions. There are few data which suggest that training with manikins has a positive influence on the treatment of real patients. Barni et al. demonstrated that in situ simulation using a high-fidelity manikin improved the management of anaphylaxis both during the acute phase and in the follow-up management of allergic patients [16]. Furthermore, Weersink et al. found a positive correlation between the assessment of residents in the simulated and workplace-based settings during resuscitations [13]. Based on the results of these studies and of the present study, it can be assumed that training with SPs has a positive effect on the treatment of real patients with a higher positive impact compared to the training with manikins.

## Conclusion

SPs are rated more realistic than manikins regarding the possibility to carry out examination techniques and taking medical history during a practical skills course in emergency medicine by undergraduate medical students. Furthermore, SPs seem to be superior to manikins with regard to teaching efficiency in an emergency medical training for undergraduate medical students.

## Data Availability

All data are available upon reasonable request.
